# Urorectal fistula repair using different approaches: operative results and quality of life issues

**DOI:** 10.1590/S1677-5538.IBJU.2020.0476

**Published:** 2021-02-03

**Authors:** Javier C. Angulo, Ignacio Arance, Yannick Apesteguy, João Felicio, Natália Martins, Francisco E. Martins

**Affiliations:** 1 Universidad Europea de Madrid Faculty of Medical Sciences Clinical Department Madrid Spain Clinical Department, Faculty of Medical Sciences, Universidad Europea de Madrid, Madrid, Spain; 2 Hospital Universitario de Getafe Madrid Spain Hospital Universitario de Getafe, Madrid, Spain; 3 Hospital das Forças Armadas Division of Urology Lisboa Portugal Division of Urology, Hospital das Forças Armadas, Lisboa, Portugal; 4 Hospital de Santa María Department of Urology Lisboa Portugal Department of Urology, Hospital de Santa María, Lisboa, Portugal

**Keywords:** complications [Subheading], Quality of Life, Fistula

## Abstract

**Purpose:**

To evaluate efficacy of urorectal fistula (URF) repair using different approaches and the clinical factor determinant of success, and also the morbidity associated to the procedure and health-related quality of life (HRQoL) in male survivors of pelvic malignancies.

**Material and Methods:**

Retrospective evaluation of 39 patients with URF primarily intervened in three institutions using different surgical approaches. Success was defined as effective fistula closure. Variables evaluated included demographics, previous treatments, surgical approach, ancillary surgeries, complications and HRQoL by using a standardized non-validated specific questionnaire. Median follow-up from surgery to interview was 55 months (interquartile range 49, range 4-112). Factors determinant of success were investigated using logistic regression. Safety of the procedure was evaluated by Clavien-Dindo scale. Deterioration of continence and erectile function and other HRQoL issues were evaluated.

**Results:**

Prostate cancer treatment was the predominant etiology. The success rate for fistula repair was 89.5%. The surgical approach was not related to failed repair (p=0.35) or complications (p=0.29). Factors associated with failure were complications (p=0.025), radiotherapy (p=0.03), fistula location (p=0.04) and fistula size (p=0.007). Multivariate analysis revealed fistula size was the only independent determinant of failure (OR 6.904, 1.01-47.75). Complications occurred in 46.2% and severe complications in 12.8%. The mortality related to the procedure was 2.6%. Urinary incontinence was present before repair in 26.3% and erectile dysfunction in 89.5%. Fistula repair caused de novo urinary incontinence in 7.9% and deterioration of erectile status in 44.7%. Globally 79% were satisfied after repair and only 7.9% rated HRQoL as unhappy. Trans-sphincteric approach was related to less deterioration of erectile function (p=0.003), and higher perceived satisfaction in QoL (p=0.04).

**Conclusions:**

The surgical approach elected to correct URF is not determinant of success nor of complications. Fistula size appears as independent determinant for failure. Transsphincteric approach could be advantageous over other procedures regarding HRQoL issues.

## INTRODUCTION

Urorectal fistula (URF) is an uncommon complication of male pelvic cancer treatment, including not only prostate, but also rectal and bladder neoplasia. It may be secondary to pelvic trauma and inflammatory bowel disease as well. However, by far the most frequent cause is radical prostatectomy, especially since laparoscopic approach has gained popularity in the last two decades. Most initial series of laparoscopic radical prostatectomy included several cases with URF, but this complication decreased to less than 2% after the learning curve of the procedure was passed (1–3). Other prostate cancer treatments can also result in this unpleasant complication (4). Possibly the most hazardous one is the combination of external radiotherapy and brachytherapy, with an incidence of 2.9% (3, 5). Cryotherapy or high-intensity focalized ultrasound (HIFU) may also cause URF, especially in a salvage therapy scenario (6).

Small URF can spontaneously heal with urinary diversion (7). However, most frequently this complication of male pelvic cancer treatment will require decisions such as whether to perform selective fecal diversion, the timing of intervention and the selection of the surgical approach based on both patient and fistula-related factors. Often these decisions are determined by the severity of presenting symptoms, the possibility of active pelvic sepsis at diagnosis, extent of tissue destruction from radiation or ablative energy, the status of the urethra and bladder neck, the distance of the fistula to the anal verge and fistula size (3). Magnetic resonance imaging or computerized tomography scan can be useful in identifying severe cavitation or post-radiotherapy osteomyelitis of the symphysis pubis (6, 8, 9). These findings may obviate an attempt to repair and serve as a guide for a permanent diversion strategy (10). It is well recognized that the success rate in repairing a complex fistula with radiation and/or ablative energy source is much lower than a fistula after surgery alone (4, 11, 12).

When rectal damage at the time of laparoscopic or robotic radical prostatectomy is not detected, URF may manifest as pelvic sepsis 10-14 days later, leading to colostomy (13). These fistulae are typically small and located in bladder neck or trigone and can be treated once sepsis or active infection have been cured. On the other hand, radiation or ablation induced fistulae are larger, fibrotic, and often involve the prostatic urethra. Along with effective closure they require interposing gracillis muscle flaps which can be performed through a perineal approach; but may also need other measures like salvage prostatectomy or omental flap through an abdominal route. Bladder neck contracture or rectal stenosis are compli cating conditions also related to radiotherapy (3). This variable spectrum of disease correlates to the multiplicity of techniques used. The relative rarity of the disease has polarized reconstructive surgeons regarding choice of one particular approach over another (14–16).

## MATERIAL AND METHODS

### Subjects

After institutional review board approval (A02/19 Hospital Universitario de Getafe) we retrospectively evaluated the medical records of 39 consecutive patients with URF due to male pelvic neoplasia intervened between January 2010 and December 2018 in three academic institutions by two surgical teams in Madrid and Lisbon. The main objective of the study was to evaluate efficacy of fistula repair and the clinical factors determinant of success. The secondary objective was to evaluate complications associated with fistula repair and health-related quality of life (HRQoL) regarding continence and potency in male survivors of pelvic malignancies.

Inclusion criteria were male patients with former pelvic cancer history, confirmation of fistula by a diagnostic imaging modality and willingness to receive surgical repair and acceptance of the complications of treatment through informed consent. Exclusion criteria were active pelvic malignancy that precluded reconstruction and fistula resulting from inflammatory disease. Data collected included demographics, former surgeries, etiology, diagnostic procedures, use of fecal and urinary diversion, definitive management, operative details, outcome, morbidity, mortality and follow-up. Complications were graded according to Clavien-Dindo classification system. The number of patients suffering urinary incontinence after URF repair and pad-count was also extracted from the clinical record. Some patients with moderate or severe bothersome stress urinary incontinence were treated with anti-incontinence devices. Type of device and outcomes were also investigated.

During 2019 subjects were contacted and answered a questionnaire through medical interview translated to Spanish and Portuguese from the original source (15). Questions were related to urinary symptoms, erectile function and HRQoL, and how surgery affected those issues (Appendix A). Urinary incontinence was defined as any involuntary loss of urine before and after URF repair, graded according to 24h pad-count. Urge incontinence and the need for medication was also investigated.

### Surgical techniques

Surgical technique was based on several factors: previous treatment, presence of radiation and or ablative procedures, the presence of local conditions such as prostate, distance from fistula to anal verge, fibrosis on digital exam and concomitant urethral stricture. Other circumstances such as concomitant morbidities were also considered. In no case was there evidence of active neoplasia. Approaches used were: abdominal (10.5%), abdominal-perineal (15.8%), perineal (29%), posterior trans-sphincteric (34.2%), anterior trans-sphincteric sagittal (5.3%), trans-coccygeal (2.6%) and trans-anal endoscopic microsurgery (2.6%).

Abdominal repair was performed through an abdominal midline incision with mobilization of the bladder neck and reconstruction, if necessary, in cases with contracture, trans-vesical excision of URF, closure with double layer technique and tissue interposition with omental flap through omentectomy along the right gastroepiploic arcade and preservation of the left gastroepiploic pedicle. Perineal approach in exaggerated lithotomy position using an inverse “Y”-shaped incision allows wide exposure of the urethra and rectum and enables posterior urethral reconstruction and use of various flap interpositions including gracillis muscle, gluteus maximum muscle, dartos and omentum if a combined abdominal-perineal approach is used. For trans-sphincteric approach the patient is placed in prone jackknife position with buttocks spread wide by adhesive tape. A midline incision is made at 11-hour lateral to the coccyx to the anal verge for a posterior trans-sphincteric approach that transects the rectal sphincter and allows endorectal open exposure. Interposition cannot be accomplished using this approach, but its anterior trans-sphincteric sagittal modification allows simultaneous reparation of the posterior urethral stricture and to use gracillis muscle flap. Careful tagging of the sphincteric muscle components and closure of the trans-sphincteric incision allows reconstruction of the anal sphincter. Trans-coccygeal approach, used for a lateral access to the rectum and bladder in URF distal to the anal verge, avoids abdominal access and allows sphincter preservation. Trans-anal endoscopic microsurgery with a specific platform is used to dissect rectal mucosa, expose proper muscle layer for suturing and create a rectal flap to cover the fistula. This technique does not address the urological part of the problem and can be used in cases with small, ncomplicated URF.

A transurethral catheter was left in situ for 3 weeks until a voiding cystourethrogram was performed to confirm anastomotic healing; the catheter was removed in the absence of extravasation. When extravasation was present, a suprapubic catheter was placed and cystogram repeated one month later. Before stoma closure another radiological investigation with barium enema was performed. In cases with colonic cancer a complete colonoscopy was also obtained before closure for better evaluation of proximal colon.

### Statistical Analysis

Median values and interquartile range (IQR) were calculated for quantitative variables and qualitative ones were described using absolute and relative frequencies. Paired t-test was used to compare continuous variables. Chi-square and Fisher exact test were performed to compare differences between data to achieve success in URF closure and to evaluate the questionnaire. Multivariate analysis of outcomes was effected with logistic regression using 95% Wald confidence intervals for the variables investigated. Statistical analysis was developed using Statistical Analysis System 9.4 (SAS Institute Inc, Cary, NY).

## RESULTS

One patient was excluded from analysis, as he died of complications, apparently with the fistula closed, but without confirmation. Median follow-up was 55 (IQR 49, range 4-112) months and patient age 62 (IQR 12.5, range 54-75) years. Nine patients (23.7%) had previous failed surgery for URF in other institutions (mean number of surgeries 1.2±0.8; range 1-3). Table-1 shows main characteristics of URF (etiology, distance to the anal verge and size), adjunct measures taken and surgical approach chosen.

**Table 1:** Main characteristics of patients (n=38) #.

Mean age, years, median ± IQR		62 ± 12.5
**Previous intervention, n (%)**		8 (21.1)
**Etiological factors, n (%)**		
	Laparoscopic radical prostatectomy	19 (50)
	Open radical prostatectomy	5 (13.2)
	Laparoscopic radical cystectomy with neobladder	2 (5.3)
	Anterior rectal resection	3 (7.9)
	Radiotherapy	15 (39.5)
	Brachytherapy	6 (15.8)
	Other ablative sources	2 (5.3)
**Distance of fistula to anal verge, n (%)**		
	< 2 cm	8 (21.1)
	2-4 cm	14 (36.8)
	4-6 cm	10 (26.3)
	> 6 cm	6 (15.8)
**Fistula size, n (%)**		
	< 1 cm	22 (57.9)
	1-2 cm	12 (31.6)
	>2 cm	4 (10.5)
**Adjunct measures taken, n (%)**		
	Fecal diversion	31 (81.6)
	Tissue interposition	14 (36.8)
	Simultaneous posterior urethroplasty	4 (10.5)
	Vesicourethral re-anastomosis	3 (7.9)
	Urinary upper tract stenting	24 (63.2)
**Surgical approach elected, n (%)**		
	Abdominal	4 (10.5)
	Abdominal-perineal	6 (15.8)
	Perineal	11 (29)
	Posterior transphincteric	13 (34.2)
	Anterior transphincteric sagittal	2 (5.3)
	Transcoccygeal	1 (2.6)
	Transanal endoscopic microsurgery	1 (2.6)

# One patient deceased during admission was excluded; IQR: interquartile range

Table-2 shows operative data, postoperative complications and long-term outcomes. The success rate for fistula repair was 89.5%. Complications occurred in 46.2% and severe complications in 12.8%. Mortality related to the procedure was 2.6%. Surgery failed in 4 patients (1 abdominal approach, 2 perineal and 1 trans-coccygeal). The surgical approach used was not associated with failure (p=0.35), nor was the number of postoperative complications (p=0.29). Measures taken after failed URF repair were pelvic exenteration (1 case), cystectomy (2 cases) and permanent ne-phrostomy (1 case). A complete list of complications is presented as supplementary material (Appendix B).

**Table 2:** Operative data, complications and outcomes (n=39) #.

Hospital admission, days, median ± IQR		9.8 ± 5
**Operative complications, n (%)#**		18 (46.2)
**Clavien-Dindo scale, n (%) #**		
	0	21 (53.8)
	I	2 (5.1)
	II	11 (28.2)
	III	1 (2.6)
	IV	3 (7.7)
	V	1 (2.6)
**Perioperative mortality rate, n (%)**		1 (2.6)
**Fistula outcome, n (%)**		
	Resolution without operation	0 (0)
	Resolution with operation	34 (89.5)
	Non-healed fistula	4 (10.5)
	Fistula recurrence after healing	0 (0)
**Fecal diversion status, n (%)**		
	Stoma closure	31 (81.6)
	Permanent stoma	7 (18.4)
**Long-term urologic outcome, n (%)**		
	Urinary incontinence after fistula repair	13 (34.2)
	Erectyle dysfunction after fistula repair	34 (89.5)
	Permanent urinary diversion	4 (10.5)
	Anti-incontinence surgical devices used	9 (23.7)
**Follow-up, months, median ± IQR**		55 ± 49

# One patient with underlying cirrhosis deceased during admission was included for complications and mortality, but was excluded for hospital admission and long-term urologic outcomes; IQR: Interquartile range

**Figure 1:**
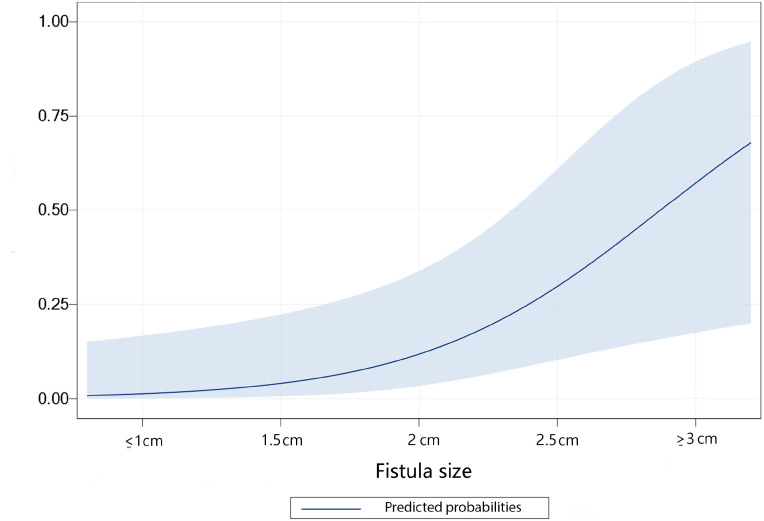
Predicted probabilities for failure to repair urorectal fistula, according to fistula size expressed in cms.

Factors associated with failed repair were fistula size (p=0.007), presence of postoperative complications (p=0.025), radiotherapy (p=0.03) and distance to anal verge (p=0.04). Factors not associated to failure were: center in which the patient was intervened (p=1.0), severity of complications (p=0.49), previous failed URF surgery (p=1.0), previous fecal diversion (p=0.8), type of fecal diversion (p=1.0), use of tissue interposition (p=0.6) or type of flap (gracillis or epi-plon) (p=5). Due to excessive correlation between variables and limited sample size, multivariate analysis revealed fistula size as the only independent determinant for failure (OR 6.9, 1.01-47.75) (Figure-1).

According to the patient questionnaire evaluated, urinary incontinence was present before fistula repair in 26.3% (10 cases) and fistula repair caused de novo urinary incontinence in 7.9% (3 cases). Urge incontinence was present in 39.5% (15 cases) cases) as mainly satisfied and only 7.9% (3 cases) as unhappy. Globally 26.3% (10 cases) consider their capacity to practice exercise limited and 79% would elect to repeat their surgery again. Once asked how URF surgery changed their QoL 32.9% (11 cases) registered great improvement, 47.4% (18 cases) improvement, 7.9% (3 cases) no change, 7.9% (3 cases) reduced and 7.9% (3 cases) severely reduced. HRQoL issues were analyzed for surgical approaches used. and medication for urge was taken by 21% (8 cases). During follow-up, 23.7% (9 cases) were treated with anti-incontinence devices (all with pad-count >3 pads/day): 5 with artificial urinary sphincter and 4 with adjustable transobturator male system. Deterioration of erectile status after URF repair took place in 44.7% (17 cases). Some degree of erectile dysfunction was present in 89.5% (34 cases): 10.5% rated their erection as enough for intercourse (4 cases), 34.2% severely deteriorated (13 cases), 15.8% almost absent (6 cases) and 39.5% totally absent (15 cases). Regarding urinary continence, some degree of urinary incontinence was present in 34.2% (13 cases). Mean pad-count was 3±2.5 (range 1-6).

Twenty-one percent (8 cases) rated their QoL as excellent, 47.4% (18 cases) as satisfied, 23.7% (9 The approaches with 1 case were omitted, abdominal-perineal and abdominal were pooled together and so were posterior and anterior trans-sphincteric (Table-3). Noticeably, trans-sphincteric approach gave less deterioration of erectile function than the rest (p=0.003) and higher satisfaction (p=0.04). A tendency for higher QoL improvement was observed on the limit of statistical significance (p=0.05).

**Table 3:** Association between the surgical approaches and specific answers to questions related to HRQoL issues.

Question		Grouped surgical approaches				
Did the erectile function change in comparison to before the fistula repair?		Abdominal (*)	Perineal	Transphincteric (#)	TOTAL (n=36)	p-value
	Yes	8 (80)	6 (54.6)	2 (13.3)	16 (44.4)	0.0003
	No	2 (20)	5 (45.4)	13 (86.7)	20 (45.6)	
**How would you describe your quality of life, if it was never going to change again?**						
	Excellent	1 (9.1)	0	7 (46.7)	8 (22.2)	
	Satisfied	6 (54.5)	6 (60)	5 (33.3)	17 (47.2)	
	Mainly satisfied	4 (36.4)	2 (20)	3 (20)	9 (25)	0.039
	Dissatisfied	0	2 (20)	0	2 (5.6)	
	Very dissatisfied	0	0	0	0	
**Would you have the same surgery performed again, if required?**						
	Yes	6 (54.5)	9 (90)	14 (93.3)	29 (80.6)	0.05
	No	5 (45.5)	1 (10)	1 (6.7)	7 (19.4)	
**How did the fistula repair change your quality of life?**						
	Severely improved	1 (9.1)	1 (10)	8 (53.3)	10 (27.8)	
	Improved	6 (54.5)	6 (60)	6 (40)	18 (50)	
	Unchanged	1 (9.1)	1 (10)	1 (6.7)	3 (8.3)	0.05
	Reduced	1 (9.1)	2 (20)	0	3 (8.3)	
	Severely reduced	2 (18.2)	0	0	2 (5.6)	

(*) Abdominal combines abdominal and abdomino-perineal approaches; (#) transphincteric combines posterior and anterior transphincteric approaches

## DISCUSSION

Walk the tightrope of avoiding failed reconstruction and not overtreating is a difficult task. Some patients undergo several URF closure attempts before success. Also, success may be so variable as to depend on a single surgery or a multi-stage scenario.

Therefore, HRQoL studies of this condition are needed. Additionally, there is not a standardized single approach to manage URF, nor is there consensus as to whether fecal diversion and other adjunct measures are always necessary. Some studies have done their best to describe an algorithm for URF management (3, 17, 18) but a classification or staging of URF in terms of complexity is still lacking.

There is a common assumption that radiotherapy and ablative procedures cause extensive tissue damage and that interposition in those cases after fistula resection and suturing of both the urinary tract and rectum is absolutely mandatory (6, 11, 16, 19). However, a “simple” approach like posterior transphincteric or even by trans-anal endoscopic microsurgery can be similarly effective without damaging fecal continence (17, 20–24). Also, the York-Mason repair may succeed even in repeated scenario (22, 24, 25), but not in all series (26, 27). A trans-anal endoscopic approach is less invasive and can be used in simple cases (e.g. very small fistula after primary laparoscopic prostatectomy without radiation), but this procedure is most often performed by experienced proctologists and is beyond the armamentarium of reconstructive urologists (28, 29). The use of rectal flap after fistula closure is the main characteristic of a trans-anal approach, either trans-sphincteric open or sphincter-sparing endoscopic, and in the absence of hypoxia or fibrosis may be sufficient for an effective closure in selected cases (17). We confirm that the modification to York-Mason using an anterior sagittal approach (anterior trans-anal trans-sphincteric sagittal, ASTRA) used by Castillo et al. allows simultaneous posterior urethroplasty and gracillis muscle flap in a prone position (2). This is a very interesting possibility that merits further exploration by experienced reconstructive urologists and permits the treatment of more complicated cases than posterior or classic York-Mason.

**Figure 2:**
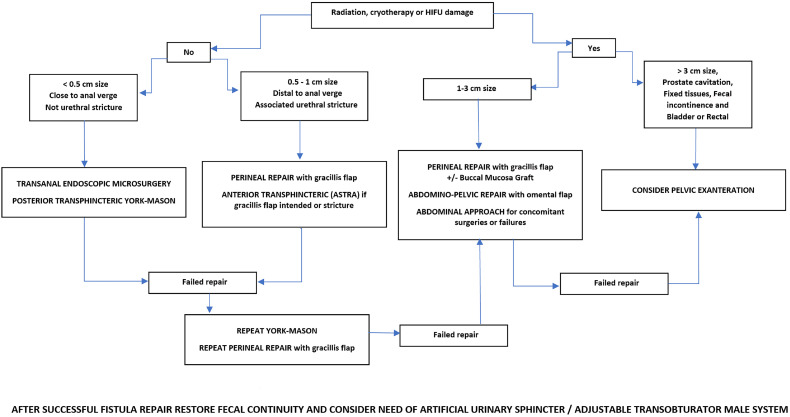
Therapeutic algorithm followed for repair of urorectal fistula.

Our primary intention was gathering the 10-years expertise of our surgical teams and identify factors predictive of treatment failure. We sought to prove whether one surgical approach was more effective than another, but we could not accomplish this objective mainly due to the relatively low failure rate and the limited number of patients. Additionally, elevated correlation between the variables (radiotherapy, fistula size, complications, distance to anal verge) included in the regression model was confounding. We could only identify fistula size as a marker of increased failure. Of course, this could be a surrogate of fistula complexity determined by the use of previous radiotherapy and ablative energy sources. Repair of URF in our hands using different surgical approaches achieved a global success rate is 89.5%. Use of therapeutic algorithms like the one we followed (Figure-2) can help choose the most appropriate technique in each different case. This and referral to high-volume institutions facilitate optimal results (3, 16, 30). Complications could also be reduced by referral. However, the rate of complications has been rarely reported (31). In our hands the risk of postoperative complications is high, with 46.2% total and 12.8% major complications.

Our second goal was the evaluation of HR-QoL in these patients. Even if the fistula is successfully repaired, patients may have persistent urinary dysfunction resulting in decreased QoL (32). A very interesting study evaluated fecal outcome measures in a small population of patients homogeneously treated with trans-perineal repair and gracillis muscle interposition and revealed bowel outcomes were better than urinary ones (33). The questionnaire we used allowed investigation of erectile dysfunction and urinary incontinence in another series (15). Again, the specific surgical approach did not seem important as there were good results in that cohort with different approaches and different types of tissue interposition. Contrary to our results these authors did not prove a worsening of erectile dysfunction due to URF repair. However, although the proportion of patients with fistula repair and urinary incontinence could be very variable, they reported incontinence in 83% of the patients specifically questioned and it was moderate--to-severe in 41.6% (15).

Using the same questionnaire, we described urinary incontinence in 34.2% and continence deterioration after URF repair in 7.9%. Erectile dysfunction occurred in 89.5% and some degree of deterioration that could be attributed to fistula repair was registered in 44.7%. Comparing surgical approaches, trans-sphincteric access caused less deterioration of erectile status than perineal and abdominal approaches. Also, a higher patient reported satisfaction was registered with this approach, which could be explained by the fact that avoiding the lateral pelvic and pararectal space dissection consistently preserves the posterolateral rectal innervations, possibly benefiting urinary continence and potency (30).

The main limitations of this study are the relative small number of patients included, the long period for patient recruiting, the retrospective nature of a part of the study and the use of a non-validated questionnaire to evaluate QoL issues prospectively during follow-up. However, this tool we use has been previously evaluated in patients with urorectal fistula with interesting results and is useful for patient evaluation. We could not ascertain how much radiation use or complexity of surgery - and not the approach itself - affect the results obtained in the questionnaire, and this is an added limitation. It would be desirable that future studies with larger numbers compare this tool with others already validated to evaluate erectile dysfunction, urinary continence and patient's perception of their quality of life.

## CONCLUSIONS

Our success rate of urorectal fistula repair was high. However, treatment morbidity is also high and QoL issues are still important in these patients. The surgical approach elected to correct URF does not appear to determine neither success nor complications. Several factors appear to be related to failure, including radiotherapy, presence of complications, longer distance to anal verge and larger fistula size. According to our casuistry, fistula size appears the only independent determinant for failure. Trans-sphincteric approach could be advantageous over perineal and abdominal routes regarding HRQoL issues. Of course, these findings should be confirmed in more robust prospective studies but, in the meantime, we should not consider York-Mason or ASTRA procedures neglectable nor obsolete. We also confirm that, in our experience, these procedures do not cause stool incontinence.

## Appendix A Patient Questionnaire used.

**Table:**

Page 1 / 2	
**1 - Do you have erections?**	
	() Yes, normal rigidity
	() Yes, slightly reduced rigidity
	() Yes, severely reduced rigidity
	() No, no erections
**2 - Did the erectile function change in comparison to before the fistula repair?**	
	() Yes
	() No
**3 - Do you involuntarily lose urine (incontinence)?**	
	() Yes
	() No
**4 - How many incontinence pads do you need per day?**	
	() None
	() No more than one
	() No more than two
	() No more than three
	() More than three
	() Security pad (no more than one)
**5 - If more than three: how many?**	
_________________________________	
_________________________________	
**6 - Do you need to void as soon as possible, once you feel the need to empty your bladder (urgency)?**	
	() Never
	() Rarely
	() Sometimes
	() Often
	() Always
**7 - Do you take any medication for urinary urgency?**	
	() Yes
	() No
**8 - As how bothersome would you describe your urinary situation to affect your life?**	
	() Not at all
	() A little
	() Bothering
	() Very bothering
**9 - How would you describe your quality of life, if it was never going to change again?**	
	() Excellent
	() Satisfied
	() Mainly satisfied
	() Dissatisfied
	() Very dissatisfied

## Appendix A Patient Questionnaire.

**Table:**

Page 2 / 2	
**10 - How much does the fistula repair limit your ability to perform sports?**	
	() Not at all
	() A little
	() Limited
	() Very limited
**11. Would you have the same surgery performed again, if required?**	
	() Yes
	() No
**12 - How did the fistula repair change your quality of life?**	
	() Severely improved
	() Improved
	() Unchanged
	() Reduced
	() Severely reduced
**13 - How satisfied are you with the surgery?**	
	() Very satisfied
	() Satisfied
	() Undecided
	() Dissatisfied
	() Very dissatisfied
**14 - If you are dissatisfied or very dissatisfied with the surgery - why?**	
	() Fistula recurrence
	() No fistula recurrence but different problem
	() Fistula recurrence and different problem
**15. Which different problems did occur?**	
________________________________	
________________________________	

## Appendix B Postoperative complications within 90 days (n=39).

**Table:**

Patient #	Grade*	Description
1	II	Urinary infection treated with antibiotics
4	II	Postoperative transfusion
5	III a	Wound dehiscence and infection intervened under local anesthesia
6	II	Postoperative ileum and transfusion
7	II	Pneumonia needing antibiotics
9	IV a	Pulmonary thromboembolism needing ICU admission
13	IV a	Renal insufficiency needing ICU admission and dialysis
15	II	Metabolic acidosis and use of total parenteral nutrition
18	II	Surgical wound infection needing antibiotics
20	II	Cardiac insufficiency treated with diuretics and cardiotropic agents
22	II	Postoperative transfusion
24	I	Postoperative ileus treated with nasogastric tube
29	II	Venous thrombosis needing anticoagulation
31	IV a	Cardiac insufficiency needing ICU admission
34	V	Death due to liver insufficiency in chronic cirrhosis
35	II	Intraoperative and postoperative transfusion
36	II	Postoperative transfusion
38	I	Scrotal hematoma treated conservatively

1 # numbered as registered in the CRF;

* According to Clavien-Dindo classification; ICU intensive care unit

## References

[1] 1. Elliott SP, McAninch JW, Chi T, Doyle SM, Master VA. Management of severe urethral complications of prostate cancer therapy. J Urol. 2006; 176(6 Pt 1):2508-13. 10.1016/j.juro.2006.07.152 17085144

[2] 2. Castillo OA, Bodden EM, Vitagliano GJ, Gomez R. Anterior transanal, transsphincteric sagittal approach for fistula repair secondary to laparoscopic radical prostatectomy: a simple and effective technique. Urology. 2006; 68:198-201. 10.1016/j.urology.2006.04.002 16806424

[3] 3. Keller DS, Aboseif SR, Lesser T, Abbass MA, Tsay AT, Abbas MA. Algorithm-based multidisciplinary treatment approach for rectourethral fistula. Int J Colorectal Dis. 2015; 30:631-8. 10.1007/s00384-015-2183-0 25808012

[4] 4. Beddy D, Poskus T, Umbreit E, Larson DW, Elliott DS, Dozois EJ. Impact of radiotherapy on surgical repair and outcome in patients with rectourethral fistula. Colorectal Dis. 2013; 15:1515-20. 10.1111/codi.12350 23841640

[5] 5. Hanna JM, Turley R, Castleberry A, Hopkins T, Peterson AC, Mantyh C, et al. Surgical management of complex rectourethral fistulas in irradiated and nonirradiated patients. Dis Colon Rectum. 2014; 57:1105-12. 10.1097/DCR.0000000000000175 25101607

[6] 6. Mundy AR, Andrich DE. Urorectal fistulae following the treatment of prostate cancer. BJU Int. 2011; 107:1298-303. 10.1111/j.1464-410X.2010.09686.x 20883482

[7] 7. Venkatesan K, Zacharakis E, Andrich DE, Mundy AR. Conservative management of urorectal fistulae. Urology. 2013; 81:1352-6. 10.1016/j.urology.2012.10.040 23528912

[8] 8. Wignall TA, Carrington BM, Logue JP. Post-radiotherapy osteomyelitis of the symphysis pubis: computed tomographic features. Clin Radiol. 1998; 53:126-30. 10.1016/s0009-9260(98)80059-7 9502089

[9] 9. Martins FE, Martins NM, Pinheiro LC, Ferraz L, Xambre L, Lopes TM. Management of iatrogenic urorectal fistulae in men with pelvic cancer. Can Urol Assoc J. 2017; 11:E372-E378. 10.5489/cuaj.4427 PMC5798442 29382460

[10] 10. Linder BJ, Umbreit EC, Larson D, Dozois EJ, Thapa P, Elliott DS. Effect of prior radiotherapy and ablative therapy on surgical outcomes for the treatment of rectourethral fistulas. J Urol. 2013; 190:1287-91. 10.1016/j.juro.2013.03.077 23538238

[11] 11. Voelzke BB, McAninch JW, Breyer BN, Glass AS, Garcia-Aguilar J. Transperineal management for postoperative and radiation rectourethral fistulas. J Urol. 2013; 189:966-71. 10.1016/j.juro.2012.08.238 PMC3570634 23009867

[12] 12. Sakai Y, Komai Y, Saito N, Ito M, Sakuraba M. Analysis of a Surgical Treatment for Persistent Urorectal Fistulas after Radical Cancer Surgery: A Comparison of Prostate Cancer and Rectal Cancer. Urol Int. 2017; 99:56-62. 10.1159/000457835 28231570

[13] 13. Chun L, Abbas MA. Rectourethral fistula following laparoscopic radical prostatectomy. Tech Coloproctol. 2011; 15:297-300. 10.1007/s10151-011-0710-8 21720888

[14] 14. Hechenbleikner EM, Buckley JC, Wick EC. Acquired rectourethral fistulas in adults: a systematic review of surgical repair techniques and outcomes. Dis Colon Rectum. 2013; 56:374-83. 10.1097/DCR.0b013e318274dc87 23392154

[15] 15. Pfalzgraf D, Isbarn H, Reiss P, Meyer-Moldenhauer WH, Fisch M, Dahlem R. Outcomes after recto-anastomosis fistula repair in patients who underwent radical prostatectomy for prostate cancer. BJU Int. 2014; 113:568-73. 10.1111/bju.12254 24053507

[16] 16. Harris CR, McAninch JW, Mundy AR, Zinman LN, Jordan GH, Andrich D, et al. Rectourethral Fistulas Secondary to Prostate Cancer Treatment: Management and Outcomes from a Multi-Institutional Combined Experience. J Urol. 2017; 197:191-4. 10.1016/j.juro.2016.08.080 27544625

[17] 17. Chen S, Gao R, Li H, Wang K. Management of acquired rectourethral fistulas in adults. Asian J Urol. 2018; 5:149-54. 10.1016/j.ajur.2018.01.003 PMC6032817 29988864

[18] 18. Choi JH, Jeon BG, Choi SG, Han EC, Ha HK, Oh HK, et al. Rectourethral fistula: systemic review of and experiences with various surgical treatment methods. Ann Coloproctol. 2014; 30:35-41. 10.3393/ac.2014.30.1.35 PMC3953168 24639969

[19] 19. Vanni AJ, Buckley JC, Zinman LN. Management of surgical and radiation induced rectourethral fistulas with an interposition muscle flap and selective buccal mucosal onlay graft. J Urol. 2010; 184:2400-4. 10.1016/j.juro.2010.08.004 20952036

[20] 20. Wood TW, Middleton RG. Single-stage transrectal transsphincteric (modified York-Mason) repair of rectourinary fistulas. Urology. 1990; 35:27-30. 10.1016/0090-4295(90)80007-a 2296812

[21] 21. Renschler TD, Middleton RG. 30 years of experience with York-Mason repair of recto-urinary fistulas. J Urol. 2003; 170(4 Pt 1):1222-5; discussion 1225. 10.1097/01.ju.0000082013.58783.17 14501729

[22] 22. Kasraeian A, Rozet F, Cathelineau X, Barret E, Galiano M, Vallancien G. Modified York-Mason technique for repair of iatrogenic rectourinary fistula: the montsouris experience. J Urol. 2009; 181:1178-83. 10.1016/j.juro.2008.10.160 19152921

[23] 23. Hadley DA, Southwick A, Middleton RG. York-Mason procedure for repair of recto-urinary fistulae: a 40-year experience. BJU Int. 2012; 109:1095-8. 10.1111/j.1464-410X.2011.10472.x 22035175

[24] 24. Bergerat S, Rozet F, Barret E, da Costa JB, Castro A, Dell'oglio P, et al. Modified York Mason technique for repair of iatrogenic recto-urinary fistula: 20 years of the Montsouris experience. World J Urol. 2018; 36:947-54. 10.1007/s00345-018-2212-z 29442154

[25] 25. Yeti ir F, arer AE, Acar HZ, Parlak O, Osmanoglu G, Karalova G. Management of Recurrent Rectourethral Fistula by York Mason Posterior Transrectal Transsphincteric Approach. Case Rep Urol. 2015; 2015:854365. 10.1155/2015/854365 PMC4684861 26770864

[26] 26. Falavolti C, Sergi F, Shehu E, Buscarini M. York Mason procedure to repair iatrogenic rectourinary fistula: our experience. World J Surg. 2013; 37:2950-5. 10.1007/s00268-013-2199-y 24045963

[27] 27. Theveniaud PE, Zafar N, Hajj AE, Germain A, Brunaud L, Eschwege P, et al. Mid term functional results following surgical treatment of recto-urinary fistulas postprostate cancer treatment. Prog Urol. 2018; 28:915-20. 10.1016/j.purol.2018.07.286 30213561

[28] 28. Kanehira E, Tanida T, Kamei A, Nakagi M, Iwasaki M, Shimizu H. Transanal endoscopic microsurgery for surgical repair of rectovesical fistula following radical prostatectomy. Surg Endosc. 2015; 29:851-5. 10.1007/s00464-014-3737-x 25060685

[29] 29. Serra-Aracil X, Labró-Ciurans M, Mora-López L, Muñoz-Rodriguez J, Martos-Calvo R, Prats-Lopez J, et al. The Place of Transanal Endoscopic Surgery in the Treatment of Rectourethral Fistula. Urology. 2018; 111:139-44. 10.1016/j.urology.2017.08.049 28916253

[30] 30. Hanna JM, Peterson AC, Mantyh C. Rectourethral fistulas in the cancer survivor. Curr Opin Urol. 2014; 24:382-8. 10.1097/MOU.0000000000000073 24841377

[31] 31. Kaufman DA, Zinman LN, Buckley JC, Marcello P, Browne BM, Vanni AJ. Short- and Long-term Complications and Outcomes of Radiation and Surgically Induced Rectourethral Fistula Repair With Buccal Mucosa Graft and Muscle Interposition Flap. Urology. 2016; 98:170-5. 10.1016/j.urology.2016.06.065 27538801

[32] 32. Raup VT, Eswara JR, Geminiani J, Madison K, Heningburg AM, Brandes SB. Gracilis muscle interposition flap repair of urinary fistulae: pelvic radiation is associated with persistent urinary incontinence and decreased quality of life. World J Urol. 2016; 34:131-6. 10.1007/s00345-015-1597-1 26008116

[33] 33. Samplaski MK, Wood HM, Lane BR, Remzi FH, Lucas A, Angermeier KW. Functional and quality-of-life outcomes in patients undergoing transperineal repair with gracilis muscle interposition for complex rectourethral fistula. Urology. 2011; 77:736-41. 10.1016/j.urology.2010.08.009 21377021

